# Safety and efficacy of same-day discharge for premature ventricular complex ablations

**DOI:** 10.1093/europace/euae205

**Published:** 2024-08-01

**Authors:** Mathew S Padanilam, Parin J Patel, Sandeep A Joshi, Girish V Nair, Bradley A Clark, Ankur Shah, Justin Field, Eric N Prystowsky, Jasen L Gilge

**Affiliations:** Department of Internal Medicine, University of Chicago, 5841 South Maryland Avenue, MC 7082, Chicago, IL 60637, USA; Department of Internal Medicine, Division of Cardiology, Ascension St Vincent, 8333 Naab Road, #400, Indianapolis, IN 46260, USA; Department of Internal Medicine, Division of Cardiology, Ascension St Vincent, 8333 Naab Road, #400, Indianapolis, IN 46260, USA; Department of Internal Medicine, Division of Cardiology, Ascension St Vincent, 8333 Naab Road, #400, Indianapolis, IN 46260, USA; Department of Internal Medicine, Division of Cardiology, Ascension St Vincent, 8333 Naab Road, #400, Indianapolis, IN 46260, USA; Department of Internal Medicine, Division of Cardiology, Ascension St Vincent, 8333 Naab Road, #400, Indianapolis, IN 46260, USA; Department of Internal Medicine, Division of Cardiology, Ascension St Vincent, 8333 Naab Road, #400, Indianapolis, IN 46260, USA; Department of Internal Medicine, Division of Cardiology, Ascension St Vincent, 8333 Naab Road, #400, Indianapolis, IN 46260, USA; Department of Internal Medicine, Division of Cardiology, Ascension St Vincent, 8333 Naab Road, #400, Indianapolis, IN 46260, USA

**Keywords:** Ablations, Ventricular arrhythmias, Electrophysiology

## Abstract

**Aims:**

Patients undergoing catheter ablation (CA) of ventricular arrhythmias (VAs) are generally observed overnight in the hospital given the concern for complications. To evaluate the efficacy and safety of same-day discharge (SDD) of patients undergoing elective CA of premature ventricular complexes (PVCs).

**Methods and results:**

A retrospective evaluation of all patients undergoing elective VA ablation at Ascension St Vincent Hospital from 1 January 2018 to 31 December 2019 was undertaken. Of those, the patients undergoing PVC ablation were divided into SDD and non-SDD. Patients underwent SDD at the discretion of the operator. The primary safety outcome was the 30-day incidence of complications and death. The primary efficacy outcome was procedural success. Among 188 patients who underwent VA ablation, 98 (52.1%) were PVC ablations, and of those, 55 (56.1%) were SDD. There was no difference in age, gender, comorbidities, or ejection fraction between the two groups. Patients that were non-SDD were more likely to be on chronic anticoagulation (*P* = 0.03), have ablation in the LV (*P* = 0.04), have retrograde access (*P* = 0.03), and receive heparin during the procedure (*P* = 0.01). There were no complications in the SDD group compared with one (2.3%) in the non-SDD group. There was no difference in primary efficacy between the two groups with a 90.9% acute success in the SDD and 88.4% in the non-SDD (*P* = 0.68).

**Conclusion:**

Same-day discharge for CA of PVCs is feasible and could lower healthcare resource utilization without compromising outcomes in this unique population.

## Graphical Abstract

**Graphical Abstract:**
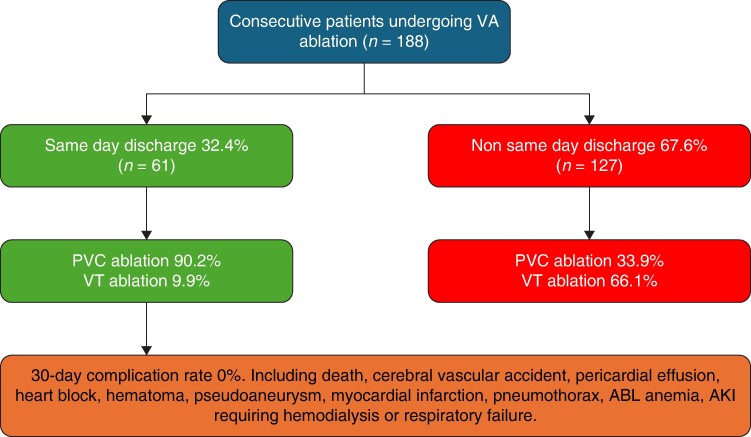

**What’s new?**

- Same-day discharge can be achieved in a select low-risk group of patients undergoing catheter ablations for ventricular arrhythmias without sacrificing safety or efficacy.
- Safe and effective same-day discharge of many patients after ventricular ablation is possible if appropriate candidates are identified.

## Introduction

Ventricular arrhythmias (VAs) may manifest as premature ventricular complex (PVC) and ventricular tachycardia (VT). Multiple studies have demonstrated the superiority of catheter ablation (CA) to antiarrhythmic therapy for the treatment of VA, and CA is increasingly utilized for the management of VAs over the past two decades.^1–8^ Due to concern of arrhythmogenicity and other potential complications, the HRS/EHRA/APHRS/LAHRS expert consensus statement recommends observing most patients undergoing VAs for at least 24 h, except for select uncomplicated right-sided ablations.^9^ The implementation of such guidelines at centres around the world is not clear, and surveys have demonstrated variability in practice styles.^10^ Opportunities for same-day discharge (SDD) after electrophysiology procedures can free up hospital staff and space for more critically ill patients. The success of SDD after other complex ablations, such as supraventricular tachycardia and atrial fibrillation, has already been demonstrated, but complications after ventricular ablations have been reported to be more frequent.^11–14^ We report our institutional experience with SDD for PVC ablations.

## Methods

### Study design

A retrospective review of all adult patients undergoing elective CA for PVC and VT from 1 January 2018 to 31 December 2019 was conducted at Ascension St Vincent Hospital, Indianapolis. Ablation was either performed for an idiopathic ventricular arrhythmia in the form of a PVC or ventricular tachycardia ablation in structural heart disease. After VA ablations, patients were eligible for SDD at the discretion of the provider if post-anaesthesia care criteria including ambulation, haemostasis, absence of apparent procedural complications, and stable vital signs were met. Patients were provided written discharge instructions with standard post-procedure care guidelines and follow-up appointments. Electronic medical records were reviewed for clinical characteristics including demographics, preprocedural risk factors, and 30-day adverse events. Procedure notes were reviewed for procedural details and complications.

### Procedural details

Patients were brought to the electrophysiology laboratory in a non-sedated and fasting state. After providing informed consent, conscious sedation or general anaesthesia was used at the operator’s discretion. The patients were then prepped and draped in standard sterile fashion. Femoral venous access and in some cases femoral arterial access was obtained with ultrasound guidance. When femoral arterial access was obtained, haemostasis was achieved with a closure device and a 3 h bedrest or 6 h bedrest prior to a mandated 1 h ambulatory observation period before discharge. All procedures were performed with the assistance of intracardiac echocardiography (ICE) with integration into 3D mapping and force sensing ablation catheters. High-density multipolar mapping catheters were utilized at the operator’s discretion. For right-sided VAs, femoral venous approach was utilized. For left-sided VA, either transseptal access under ICE with pressure waveforms or retrograde access via the femoral artery was obtained. If ablation was above the coronary cusps, ICE integration with 3D mapping was utilized to ensure a safe distance from the coronary arteries. Mapping of the VA was always performed with the assistance of three-dimensional electroanatomic mapping. Fluoroscopy was utilized if necessary. For PVC ablations, mapping was performed targeting the earliest bipolar signal accompanied by an early unipolar signal with a QS morphology. All ablations were performed with two to three venous access sites, a force sensing and irrigated ablation catheter. Ablation parameters varied and were performed based on each individual operator’s preference. At the end of every procedure, final ICE imaging was obtained to ensure that there was no significant pericardial effusion.

### Outcomes

A 30-day incidence of complications including death, cerebral vascular accident, pericardial effusion, heart block, haematoma, pseudoaneurysm, myocardial infarction, pneumothorax, acute blood loss requiring transfusion, acute kidney injury requiring haemodialysis, or respiratory failure requiring intubation or inability to extubate post-procedure was analysed in all patients that were discharged the same day. Acute procedural success was evaluated and defined as freedom from the clinical arrhythmia at least 30 min after ablation was performed. The same outcomes were analysed in the non-SDD group when observed overnight, or longer, post-ablation. More importantly, the baseline demographics of the two groups were compared to predict SDD.

### Statistical analysis

Descriptive statistics of the SDD and non-SDD groups were summarized using frequencies and/or means. Statistical significance of differences was established by using χ^2^ test for categorical variables and Student’s *t*-test for continuous variables. Two-tailed *P*-values of <0.05 were considered statistically significant. Tests were conducted using SPSS version 26 (IBM Corporation, Armonk, New York). All relevant data are presented here, and further information may be provided as requested.

## Results

A total of 188 CAs were performed for VAs between the dates 1 January 2018 and 31 December 2019. Premature ventricular complexes constituted 98 (52.1%) ablations, VT constituted 73 (38.8%) ablations, and ablations of both PVC and VT occurred 17 (9.0%) times. Of the 188 ablations, 61 (32.4%) were discharged the same day (SDD group). One hundred twenty-seven patients were not discharged the same day (non-SDD group). Baseline demographics and clinical characteristics of the SDD group and non-SDD group for all VA are summarized in *Table 1*. For all patients, the median age was 61.4 ± 13 and 58 (30.9%) were female. The arrhythmogenic substrate for the PVC group was idiopathic VA while the VT group was structural heart disease with either ischaemic or non-ischaemic cardiomyopathy. Idiopathic ventricular arrhythmias originated from either the outflow tract, papillary muscles, or moderator band. Femoral vein was accessed in all cases, femoral artery in 79 (43.6%) cases, and transseptal puncture in 47 (26%) cases. Ultrasound-guided vascular access and intracardiac echocardiogram were employed in all cases. Ablations were performed in right ventricle (RV) only in 66 (35.1%) cases, left ventricle (LV) only in 77 (41%) cases, and RV and LV in 45 (23.9%) cases.

**Table 1:** Baseline demographics and characteristics of the study population

	Total (*n* = 188)	SDD (*n* = 61)	Non-SDD (*n* = 127)	*P*-value
Age	61.4 ± 13.0	55.8 ± 14.4	64.1 ± 12.8	0.01
Length of stay	2.2 ± 4.3	0 ± 0	3.3 ± 4.9	0.01
Gender (female)	58 (30.9%)	24 (39.3%)	34 (26.8%)	0.08
BMI	30.5 ± 6.6	30.3 ± 6.5	30.5 ± 6.7	0.83
Antiplatelet use	114 (61.3%)	33 (55.9%)	81 (63.8%)	0.31
Anticoagulation use	28 (14.9%)	4 (6.6%)	24 (18.9%)	0.03
Beta blocker	137 (72.9%)	42 (68.9%)	95 (74.8%)	0.39
Calcium channel blocker	14 (7.5%)	6 (9.8%)	8 (6.3%)	0.39
Antiarrhythmic	78 (41.7%)	17 (28.3%)	61 (48%)	0.01
Diabetes mellitus	42 (22.5%)	9 (15%)	33 (26%)	0.09
Coronary artery disease	75 (39.9%)	20 (32.8%)	55 (43.3%)	0.17
CABG	20 (10.6%)	4 (6.6%)	16 (12.6%)	0.21
Hyperlipidaemia	100 (53.2%)	34 (55.7%)	66 (52%)	0.63
Hypertension	107 (56.9%)	31 (50.8%)	76 (59.8%)	0.24
Chronic obstructive pulmonary disease	21 (11.2%)	5 (8.2%)	16 (12.6%)	0.37
CKD	12 (6.4%)	1 (1.6%)	11 (8.7%)	0.07
CVA/TIA	6 (3.2%)	2 (3.3%)	4 (3.1%)	0.96
Congestive heart failure	67 (35.6%)	22 (36.1%)	45 (35.4%)	0.93
LVAD	5 (2.7%)	0 (0%)	5 (3.9%)	0.12
VT storm	6 (3.2%)	0 (0%)	6 (4.7%)	0.08
Atrial fibrillation	41 (21.8%)	7 (11.5%)	34 (26.8%)	0.02
LVEF (%)	41.7 ± 16.1	48.7 ± 12.7	38.2 ± 16.6	0.01

### Premature ventricular complex ablation group

Of the 188 patients undergoing CA for VA, 98 were for PVCs. The median age was 57.6 ± 13.4 years and 42 (42.9%) were female as described in *Table 2*. Common comorbidities include hypertension (HTN) (54.1%) and hyperlipidaemia (HLD) (48%). Coronary artery disease (25.5%) and congestive heart failure (23.5%) were not as common with a median LVEF of 49.3 ± 11.9%. Chronic antiplatelet use was present in 49%, and chronic anticoagulant use was present in 11.2% of patients. During the procedure, the RV was ablated in 44.9% of patients. The LV was ablated in 31.6% while both ventricles were ablated in 23.5% of cases. Transseptal access was performed in 14.7% of cases while retrograde access was performed in 44.2% of cases. When retrograde access was performed, arterial closure device was used 72.7% of the time. The median length of stay was 0.5 ± 0.9 days.

**Table 2:** Baseline demographics and characteristics for PVC ablation

	Total (*n* = 98)	SDD (*n* = 55)	Non-SDD (*n* = 43)	*P*-value
Age	57.6 ± 13.4	55.5 ± 15	60.4 ± 10.8	0.07
Length of stay	0.5 ± 0.9	0 ± 0	1.2 ± 1.1	0.01
Gender (female)	42 (42.9%)	31 (56.4%)	18 (41.9%)	0.86
BMI	30.8 ± 7.2	30.2 ± 6.8	31.7 ± 7.6	0.28
Antiplatelet use	47 (49%)	27 (50.9%)	20 (46.5%)	0.67
Anticoagulation use	11 (11.2%)	3 (5.5%)	8 (18.6%)	0.04
Beta blocker	69 (70.4%)	37 (67.3%)	32 (74.4%)	0.44
Calcium channel blocker	10 (10.2%)	6 (10.9%)	4 (9.3%)	0.79
Antiarrhythmic	24 (24.5%)	14 (25.5%)	10 (23.3%)	0.8
Diabetes mellitus	14 (14.3%)	8 (14.5%)	6 (14%)	0.93
Coronary artery disease	25 (25.5%)	15 (27.3%)	10 (23.3%)	0.65
CABG	2 (2%)	1 (1.8%)	1 (2.3%)	0.86
Hyperlipidaemia	47 (48%)	28 (50.9%)	19 (44.2%)	0.51
Hypertension	53 (54.1%)	26 (47.3%)	27 (62.8%)	0.13
Chronic obstructive pulmonary disease	5 (5.1%)	2 (3.6%)	3 (7%)	0.46
CKD	3 (3.1%)	1 (1.8%)	2 (4.7%)	0.42
CVA/TIA	1 (1%)	1 (1.8%)	0 (0%)	0.37
Congestive heart failure	23 (23.5%)	17 (30.9%)	6 (14%)	0.05
LVAD	0 (0%)	0 (0%)	0 (0%)	0.12
Atrial fibrillation	15 (15.3%)	6 (10.9%)	9 (20.9%)	0.17
LVEF (%)	49.3 ± 11.9	50.2 ± 12.1	48.2 ± 11.7	0.43
Transseptal access	14 (14.7%)	8 (14.5%)	6 (15%)	0.95
Retrograde access	42 (44.2%)	19 (34.5%)	23 (57%)	0.03
Arterial closure	40 (72.7%)	19 (34.5%)	21 (63.6%)	0.06
Ventricle ablated				0.04
Right ventricle	44 (44.9%)	30 (54.5%)	14 (32.6%)	
Left ventricle	31 (31.6%)	12 (21.8%)	19 (44.2%)	
Biventricular	23 (23.5%)	13 (23.6%)	10 (23.3%)	
Heparin use	63 (64.6%)	25 (46.3%)	38 (88.4%)	0.01

### Same-day discharge in premature ventricular complex ablation group

Fifty-five patients had a PVC ablation and were discharged the same day. The median age was 55.5 ± 15 years, and 31 (56.4%) were female. No patient stayed overnight for observation, and all patients were performed as outpatients. The two most common comorbidities in this group include hyperlipidaemia (50.9%) and hypertension (47.3%) while coronary artery disease (27.3%) and congestive heart failure (30.9%) were not as common. The median LVEF was 50.2 ± 12.1%. Antiplatelets were taken by 50.9% of patients while only 5.5% were on chronic anticoagulation. The majority of ablations was performed in the right ventricle (54.5%) with retrograde access being performed in 34.5% and transseptal access in 14.5% for LV ablation. Heparin was administered in 46.3% of procedures. There were no complications in this group, and procedural success occurred in 50 (90.9%) cases.

### Non-same-day discharge in premature ventricular complex ablation group

Forty-three patients had a PVC ablation and were not discharged the same day. The median age was 60.4 ± 10.8 years, and 18 (41.9%) females were in the non-SDD group. The median length of stay was 1.2 ± 1.1 days. Similar to the SDD group, the most common comorbidities included hypertension (62.8%) and hyperlipidaemia (44.2%). Coronary artery disease (23.3%) and congestive heart failure (14%) were also common with a median LVEF of 48.2 ± 11.7. Antiplatelets were taken by 46.5% of patients while 18.6% were on chronic anticoagulation. The left ventricle was the most common site of ablation (44.2%). Retrograde access was performed in 57% of patients with 15% receiving transseptal access. Acute procedural success was achieved in 38 (88.4%) with a complication rate of 2.3% due to a single episode of complete heart block. The average length of stay was 1.2 ± 1.1 days.

### Comparison of groups

There were no differences in age, sex, BMI, or comorbidities between the two groups. There was a higher rate of chronic anticoagulation in the non-SDD group (*P* = 0.01) but no difference in antiplatelet or antiarrhythmic use. Patients that were discharged the same day had a higher rate of RV ablation while those that were observed overnight had a higher rate of LV ablation (*P* = 0.04). Retrograde access was also performed more in those that were observed overnight (*P* = 0.03). Heparin was administered at a higher rate in those that were not discharged the same day (*P* = 0.01). There was no difference in procedural success between the two groups (90.9% vs. 88.4%; *P* = 0.68).

### Adverse events in the premature ventricular complex ablation group

No adverse events or rehospitalizations were experienced within the 30-day post-operative period for the 55 patients in the SDD group. One (2.3%) patient in the non-SDD group experienced a peri-procedural complication (*Table 3*). There were no 30-day rehospitalizations in either the SDD group or the non-SDD group. There were no intraprocedural or 30-day mortalities. Heart block occurred in one patient that did require prolonged hospitalization and pacemaker implantation. No other complication occurred.

**Table 3:** Adverse events 30 days post-PVC ablation

	Total (*n* = 98)	SDD (*n* = 55)	Not SDD (*n* = 43)
Any adverse event	1 (1%)	0 (0%)	1 (2.3%)
CVA	0 (0%)	0 (0%)	0 (0%)
Mortality	0 (0%)	0 (0%)	0 (0%)
In lab mortality	0 (0%)	0 (0%)	0 (0%)
Pericardial effusion	0 (0%)	0 (0%)	0 (0%)
Heart block	1 (1%)	0 (0%)	1 (2.3%)
Haematoma	0 (0%)	0 (0%)	0 (0%)
Pseudoaneurysm	0 (0%)	0 (0%)	0 (0%)
Myocardial infarction	0 (0%)	0 (0%)	0 (0%)
Pneumothorax	0 (0%)	0 (0%)	0 (0%)
Acute blood loss	0 (0%)	0 (0%)	0 (0%)
Acute kidney injury	0 (0%)	0 (0%)	0 (0%)
Respiratory failure	0 (0%)	0 (0%)	0 (0%)
Rehospitalization	0 (0%)	0 (0%)	0 (0%)

## Discussion

Our single centre experience with CA of VAs shows the feasibility of SDD in a subgroup of patients undergoing PVC ablation. Nearly one-third of the ventricular ablations (*n* = 61) conducted at our centre in the two-year span studied were discharged the same day, and these patients had no 30-day adverse events or rehospitalizations. Same-day discharge was achieved in 56.1% of patients after PVC and 5.5% of patients after VT ablations. The SDDs were at the discretion of the physician after meeting discharge criteria.

Ventricular ablations can result in myocardial inflammation and scar with concerns of potential arrhythmogenicity from the periablation areas. Patient safety in relation to potential life-threatening VAs has been the one of the concerns leading to routine admission of patients after VA ablations.^9^ Previous experience from tertiary centres reported an increase in complication rate over time with an in-hospital complication rate of up to 5.3%.^13^ This study demonstrates no life-threatening VAs post-ablation, and no patients required rehospitalization within 30 days of their procedure after PVC ablation. Utilization of technology such as ultrasound for vascular access, intracardiac ultrasound, and contact force sensing catheters may have played a role in the safety of these procedures. The safety and feasibility of SDD after complex catheter ablations have been demonstrated for atrial fibrillation and atrial flutter.^12^ To the best of our knowledge, this is the first study evaluating the safety and efficacy of SDD after CA of VAs.

In a European Heart Rhythm Association survey from 2020, physicians reported ∼15% SDD after VT ablations.^14^ Variables most relevant in considering SDD in that study included frailty, ending hour of the procedure, age, recovery from sedation, duration of the procedure, and symptomatic heart failure with New York Heart Association functional class III/IV. The overall 30-day complication rate in the entire cohort was 4.3% and had a higher tendency to occur in patients with coronary artery disease, congestive heart failure, and lower ejection fractions.

While there is no definitive risk stratification tool to help identify which patients undergoing CA for VAs may safely be discharged the same day, our single centre experience over two years suggests that a portion of individuals may be discharged with low risk of complications. The operators preferentially discharged those undergoing PVC ablations as 90.2% of SDDs were PVC ablations and 9.9% included VT ablations. Despite 90.2% of SDD being PVC ablations, this only accounted for 56.1% of all PVC ablations. Chronic anticoagulation, ablation in the left ventricle, retrograde access, and administration of heparin were significantly higher in the non-SDD group. This portrays the complexity and variability in ventricular ablations and emphasizes that SDD may require individualized consideration. Larger multicentre studies may be able to further elucidate which individual criteria may predict safe and effective SDD.

## Limitations

Limitations inherent to any retrospective cohort study are applicable here including lack of a randomized control group for comparison. Physician and patient bias could have played a role in patient selection of patients for observation even when standard discharge criteria were met. The total number of adverse events across all patients is low, which limits the power of statistical analysis. However, lack of adverse events in the SDD group is reassuring. It is possible that universal use of ultrasound-guided vascular access and ICE during the procedure improved outcomes in our series, and it may not be applicable to cases where these technologies are not utilized. Future studies are needed to more precisely define the patient population that can be successfully discharged the same day.

## Conclusion

Same-day discharge for CA of PVCs is feasible in a low-risk population. This approach could lower healthcare resource utilization without compromising outcomes in this unique population.

## Data Availability

The data underlying this article will be shared on reasonable request to the corresponding author.
